# The Exocyst Is Required for CD36 Fatty Acid Translocase Trafficking and Free Fatty Acid Uptake in Skeletal Muscle Cells

**DOI:** 10.3390/cells11152440

**Published:** 2022-08-06

**Authors:** Nicole K. Nakamura, Darcy S. Tokunaga, Herena Y. Ha, Noemi Polgar

**Affiliations:** Department of Anatomy, Biochemistry, and Physiology, John A. Burns School of Medicine, University of Hawaii, Honolulu, HI 96813, USA

**Keywords:** exocyst, CD36 fatty acid translocase, skeletal muscle

## Abstract

In obesity, chronic membrane-localization of CD36 free fatty acid (FFA) translocase, but not other FFA transporters, enhances FFA uptake and intracellular lipid accumulation. This ectopic lipid accumulation promotes insulin resistance by inhibiting insulin-induced GLUT4 glucose transporter trafficking and glucose uptake. GLUT4 and CD36 cell surface delivery is triggered by insulin- and contraction-induced signaling, which share conserved downstream effectors. While we have gathered detailed knowledge on GLUT4 trafficking, the mechanisms regulating CD36 membrane delivery and subsequent FFA uptake in skeletal muscle are not fully understood. The exocyst trafficking complex facilitates the docking of membrane-bound vesicles, a process underlying the controlled surface delivery of fuel transporters. The exocyst regulates insulin-induced glucose uptake via GLUT4 membrane trafficking in adipocytes and skeletal muscle cells and plays a role in lipid uptake in adipocytes. Based on the high degree of conservation of the GLUT4 and CD36 trafficking mechanisms in adipose and skeletal muscle tissue, we hypothesized that the exocyst also contributes to lipid uptake in skeletal muscle and acts through the targeted plasma membrane delivery of CD36 in response to insulin and contraction. Here, we show that the exocyst complex is necessary for insulin- and contraction-induced CD36 membrane trafficking and FFA uptake in muscle cells.

## 1. Introduction

Insulin resistance (IR) is a prelude to type-2 diabetes and a hallmark of obesity. Its prevalence in the U.S. population is on the rise [1]. Obese patients have elevated circulating free fatty acid (FFA) levels compared to lean individuals [2]. When the skeletal muscle is challenged with excess FFA, which it cannot oxidize, the imbalance leads to intramyocellular accumulation of diacylglycerol and ceramide [3]. These lipid intermediates promote IR by inhibiting insulin-induced protein kinase B (Akt) phosphorylation and downstream GLUT4 glucose transporter trafficking and glucose uptake [4,5].

The transmembrane glycoprotein FFA translocase CD36 (cluster of differentiation 36) is responsible for metabolic stimulus-induced FFA uptake and controls subsequent fatty acid metabolism [6,7]. Skeletal muscle FFA uptake correlates with the amount of CD36 on the cell membrane, which is regulated by vesicular trafficking of CD36 between its endosomal storage compartment and the sarcolemma in response to physiological stimuli [8,9]. Chronic membrane-localization of CD36, but not other FFA transporters (FABP3, FATP1, or FATP4), enhances FFA uptake and intracellular lipid accumulation in obesity [10]. In lipid oversupply, FFA binding to CD36 initiates AMP-activated protein kinase (AMPK) signaling, triggering a positive feedback loop of further CD36 membrane delivery [11].

Recent studies proposed that CD36 could be a potential therapeutic target for IR, as CD36-mediated FFA uptake is an essential contributor to intramyocellular lipid accumulation triggering IR [12]. Transgenic modulation of CD36 expression significantly impacts lipid and glucose homeostasis, and CD36 knockout mice exhibit higher FFA and triglyceride levels with lower fasting glucose and insulin levels [13]. CD36 deficiency also improves muscle glucose uptake and insulin sensitivity [6,14]. Based on the above, it is theorized that directly decreasing CD36 translocation to the muscle cell membrane could provide a mechanism to overcome lipid accumulation leading to IR.

CD36 and GLUT4 are the predominant skeletal muscle fuel transporters regulating FFA and glucose flux, respectively. In major metabolic tissues, insulin- and contraction-induced signaling trigger convergent pathways that regulate both CD36 and GLUT4 membrane delivery via shared downstream effectors [15,16]. Chronic lipid overload has a distinct effect on CD36 and GLUT4 membrane transport: CD36 is relocated to the sarcolemma, but GLUT4 is retained in intracellular GLUT4 storage vesicles [11,17]. While we understand the molecular machinery responsible for GLUT4 trafficking, the mechanisms regulating CD36 membrane transport in skeletal muscle remain largely unknown. Identifying the shared and unique elements regulating CD36 and GLUT4 membrane transport could provide therapeutic targets to counter fatty acid overload-induced myocellular lipid accumulation and impaired glucose uptake in insulin-resistant states.

One of the factors implicated in regulating insulin-induced FFA uptake is the exocyst, which was identified in a recent study using cultured 3T3-L1 adipocytes [18]. This trafficking complex consists of eight subunits, EXOC1–8. The exocyst mediates secretory vesicle targeting and tethering to certain membrane domains, a process underlying the controlled delivery of fuel transporters. In addition, a known regulator of exocyst activity, the small GTPase Rab8a, is essential for insulin-induced CD36 trafficking and FFA uptake in muscle [19,20]. The exocyst also regulates GLUT4 trafficking in response to insulin in adipocytes [21] and skeletal muscle cells [22] and interacts with the v-SNARE vesicle-associated membrane protein-3 (VAMP-3) necessary for both GLUT4 and CD36 vesicle fusion [23,24]. Whether the exocyst-mediated delivery mechanism is also shared between GLUT4 and CD36 in skeletal muscle remains to be determined. In this study, we analyzed the role of the exocyst complex in CD36 cell surface delivery and subsequent FFA uptake in cultured skeletal muscle cells.

## 2. Materials and Methods

### 2.1. Cell Culture

L6-GLUT4myc, a myoblast cell line of rat skeletal myoblasts, was purchased from Kerafast Inc. These cells express a GLUT4 glucose transporter with a myc epitope tag on an extracellular loop. Cells were cultured in complete Minimum Essential Medium—Alpha (MEM alpha) with nucleotides supplemented with 10% fetal bovine serum (FBS) and 1% antibiotic-antimycotic solution (100 U/mL of penicillin and 100 μg/mL of streptomycin). Prior to insulin and ionomycin treatment, the cells were serum-starved in MEM alpha with 0.5% FBS for 3 h. Subsequently, they were stimulated either with 0 nM or 100 nM human recombinant insulin in Krebs Ringer Buffer (pH 7.4) with 0.1% bovine serum albumin (BSA) at 37 °C for 30 min or with 0 nM or 1 μM ionomycin in Krebs Ringer Buffer (pH 7.4) with 0.1% bovine serum albumin (BSA) at 37 °C for 10 min. Treated and respective control cells were collected in parallel under the same conditions.

### 2.2. Immunofluorescent Staining

Immunofluorescent staining of L6-GLUT4myc myoblasts was performed as previously described [22] to determine the colocalization of EXOC5 and CD36 in response to insulin and ionomycin treatment.

L6-GLUT4myc myoblasts were grown on coverslips to 70–80% confluence. Following treatment with insulin or ionomycin, the cells were fixed with 4% paraformaldehyde for 10 min at room temperature and permeabilized. After blocking with 0.1% BSA in PBS, samples were incubated overnight in primary antibodies anti-EXOC5 (Cat# sc-514802, Santa Cruz Biotechnologies, Dallas, TX, USA) and anti-CD36 (Cat# NB400-144, Novus Biologicals LLC, Centennial, CO, USA) at 4 °C. The samples were subsequently incubated with secondary antibodies (DyLight 488 Anti-Rabbit IgG and DyLight 594 Anti-Mouse, Vector Laboratories, Newark, CA, USA) at a 1:1000 dilution at room temperature for 1 h. We used DAPI as a nuclear stain and mounted the samples with Fluoromount-G mounting medium (Thermo Fisher Scientific, Waltham, MA, USA). Imaging was performed using a Leica SP8 confocal microscope (Leica Microsystems GmbH, Wetzlar, Germany).

### 2.3. Proximity Ligation Assay

As previously described, the PLA was performed using the Duolink^®^ PLA system (Sigma-Aldrich, Burlington, MA, USA) [22]. Briefly, L6-GLUT4myc myoblasts were grown on chamber slides to 70–80% confluence. Following treatment, the cells were fixed in 4% paraformaldehyde, permeabilized and blocked with a blocking buffer of 1% BSA in PBS at room temperature for 1 h. The cells were then incubated with a combination of primary antibody pairs (anti-Rab8a (Rabbit, Cat# 55296-1-AP, Proteintech, Rosemont, IL, USA), anti-Rab11 (Rabbit, Cat# 5589S, Cell Signaling Technology, Danvers, MA, USA), anti-Rab14 (Mouse, Cat# sc-271401, Santa Cruz Biotechnologies,), anti-CD36 (Rabbit, Cat# NB400-144, Novus; Mouse, Cat# 66395-1-Ig, Proteintech), anti-EXOC5 (Rabbit, Cat# 17593-1-AP, Proteintech; Mouse, Cat# sc-514802, Santa Cruz Biotechnologies) and anti-EXOC7 (Mouse, Cat# sc-365825, Santa Cruz Biotechnologies), as indicated in the figures) at 4 °C overnight. Following washes, the secondary antibody conjugate mixture (Duolink In Situ PLA probes: anti-goat plus and anti-mouse minus) was added. Upon ligation, we performed signal amplification with fluorescently labeled oligonucleotide detection probes (Duolink In Situ Detection Reagents Red, Sigma). The samples were mounted using the Duolink In Situ Mounting Medium with DAPI. Imaging was performed using a Leica SP8 confocal microscope, and signal-to-nuclei ratios were measured using ImageJ [25] by a blinded experimenter.

### 2.4. In-Cell Western to Quantify Cell-Surface Delivery of CD36 in Cultured Cells

Cell-surface delivery of CD36 was quantified as previously described. [22] Briefly, L6-GLUT4myc myoblasts were plated at a cell density of 3 × 10^5^ cells/well into 8-well chamber slides and allowed to attach overnight. Cells were then treated with insulin or ionomycin as described above in the presence of the exocyst inhibitor endosidin 2 (ES2) or DMSO as control. Heterozygous EXOC5 knockout (ΔEXOC5) cells were stimulated with 100 nM insulin or 1 μM ionomycin in parallel with the appropriate controls, as described above. Following fixation with 4% paraformaldehyde and blocking with 5% BSA in PBS, surface CD36 was detected using a primary antibody targeting the extracellular domain of CD36 (Rabbit, Cat# NB400-144, Novus) diluted 1:100 in 5% BSA in PBS. Following subsequent permeabilization in 0.1% Triton X-100 for 10 min, the cells were incubated with an anti-β-tubulin primary antibody (Mouse, Proteintech, sc-5274) diluted 1:100 as control. Cells were then incubated with IRDye secondary antibodies (LI-COR Biosciences, Lincoln, NE, USA) diluted at 1:15,000. The chamber slides were scanned using the Odyssey Clx scanner. Densitometry was then performed with Image Studio software (LI-COR Biosciences). Surface CD36 signal intensity was normalized with total β-tubulin levels measured.

### 2.5. Fatty Acid Uptake Assay

To measure FFA uptake in skeletal muscle cells, we adopted a BODIPY-palmitate protocol [26] for cultured skeletal myoblasts. L6 GLUT4myc WT cells were grown on 96-well plates overnight at a density of 1.6 × 10^4^ cells/well and, following serum-starvation, treated with insulin or ionomycin as described above. After incubation, the medium was aspirated, and cells were washed twice with PBS containing fatty acid-free albumin (0.4%) and incubated in BODIPY-conjugated palmitate (5 μM) for 2 min at 37 °C. Following incubation, treatments were aspirated and washed three times in ice-cold 1X PBS, and fluorescence from BODIPY-conjugated fatty acids was measured using a SpectraMax M3 microplate reader (Molecular Devices, LLC, Silicon Valley, CA, USA)

## 3. Results and Discussion

### 3.1. Insulin and Ionomycin Stimulate Exocyst-CD36 Colocalization

The exocyst is responsible for the targeted delivery and docking of membrane-bound secretory vesicles [27]. In cardiac and skeletal muscle cells, membrane delivery of fuel transporters GLUT4 and CD36 in response to insulin and contraction is dependent on GTPase activating protein AS160 [20] and small regulatory GTPases, such as RalA and Rab8 [28,29,30]. The Rab effector exocyst proved necessary for FFA uptake in adipocytes [18]. However, it is not known if the exocyst regulates adipocyte FFA uptake via CD36 membrane transport or if the exocyst has a similar function in skeletal muscle cells.

**Figure 1:**
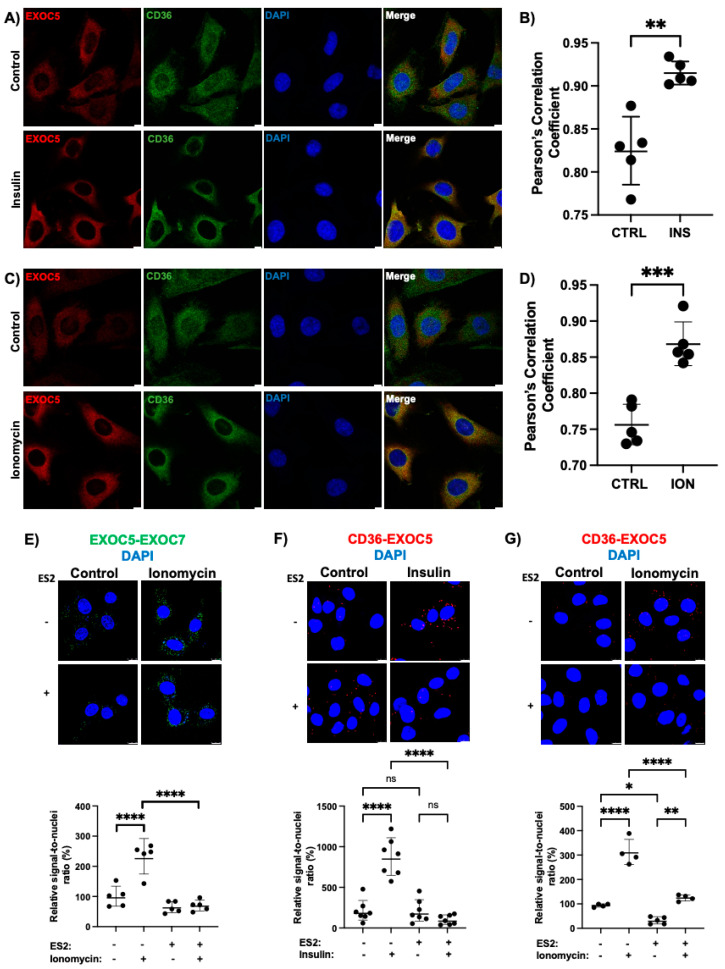
The exocyst is recruited to CD36 vesicles in response to stimuli. (**A**,**C**) Confocal images of control and insulin− or ionomycin−induced L6−GLUT4myc rat skeletal myoblasts immunostained for exocyst subunit Exoc5 and CD36 show an increased colocalization of CD36 and Exoc5 in skeletal myoblasts upon treatment. (**B**,**D**) Colocalization is expressed as average coefficients of correlation (Pearson’s). (**E**) Microscopic images and quantification of a PLA assessing EXOC5 and EXOC7 subunit proximity in L6 GLUT4myc myoblasts show an increase in exocyst subunit proximity following ionomycin treatment. In the presence of exocyst inhibitor ES2, ionomycin fails to stimulate exocyst assembly. (**F**,**G**) Microscopic images and quantification of a PLA evaluating EXOC5 subunit proximity with CD36 in L6 GLUT4myc myoblasts following insulin or ionomycin treatment in the presence or absence of ES2. Both treatments stimulate EXOC5 recruitment to CD36, as demonstrated by an increase in PLA signals—shown in red or green. The DAPI stain is blue. Images are representative of at least three independent experiments. Data are presented as mean ± S.D normalized to controls. * *p* < 0.05; ** *p* < 0.01; *** *p* < 0.005; **** *p* < 0.0005; ns: *p >* 0.05. Scale bars: 5 µm (**A**,**C**); 10 µm (**E**–**G**).

We used immunofluorescent staining to determine the impact of insulin and the contraction-mimetic Ca^2+^ ionophore ionomycin on the subcellular localization of the exocyst subunit EXOC5 and CD36. After insulin or ionomycin treatment, colocalization of CD36 and the exocyst subunit EXOC5 increased (Figure 1A,C), as supported by our Pearson’s correlation coefficient calculations, indicative of the degree of colocalization between our target proteins (Figure 1B,D). Our data are the first to suggest that the exocyst subunit EXOC5 colocalizes with the FFA translocase CD36 at the basal state in skeletal muscle cells. This colocalization is further amplified in response to stimuli. These findings agree with previous work demonstrating that certain exocyst subunits, including EXOC5, travel with the membrane-bound vesicles along the cytoskeleton to the site of vesicle fusion [31].

We have established that insulin triggers exocyst assembly in skeletal myoblasts [22]. To determine if contraction induced recruitment of the exocyst subunits, we assessed the effects of the exercise-mimetic ionomycin on complex assembly. The changes in the subcellular proximity of exocyst subunits EXOC5 and EXOC7 in response to ionomycin treatment were measured in L6 GLUT4myc skeletal myoblasts using proximity ligation assays (PLA). A PLA signal indicates physical proximity, as it is generated only when the two target proteins (i.e., EXOC5 and EXOC7) are within 40 nanometers of each other. The number of signal puncta is proportional to the number of associations between the targets interrogated [32,33]. Following quantification of signal-to-nuclei ratios in control and ionomycin-treated L6 GLUT4myc cells, we observed significantly higher association rates between the exocyst subunits EXOC5 and EXOC7 in response to ionomycin compared to vehicle-treated controls (Figure 1E). This suggests that acute ionomycin treatment, like insulin, can trigger exocyst complex assembly in L6 GLUT4myc myoblasts. In addition, treatment with the exocyst-inhibitor endosidin-2 (ES2) [34] alone did not significantly alter complex assembly compared to the baseline. However, in the presence of ES2, ionomycin did not stimulate exocyst assembly (Figure 1E). These observations suggest that ionomycin triggers exocyst complex assembly in skeletal myoblasts, and the inhibitor ES2 targeting EXOC7 hinders this process.

### 3.2. The Exocyst Is Recruited to CD36 Vesicles in Response to Stimuli

A recent study showed that the exocyst regulates insulin-induced FFA uptake in cultured adipocytes [18]. However, it remained unclear if the trafficking complex exerted its effects through cell-surface delivery of fuel transporters or via intracellular transport contributing to lipid-droplet formation. CD36 is the predominant membrane protein responsible for metabolic stimulus-induced FFA uptake in adipocytes, cardiac-, and skeletal myocytes [6]. The presence of cell surface CD36 governs the rate of FFA uptake in skeletal muscle cells. Moreover, CD36 knockout mice show decreased FFA uptake rates in vivo in the heart, skeletal muscle and adipose tissue [35], and CD36 is implicated in dysregulated FFA uptake and lipid metabolism [10].

The regulatory mechanisms of CD36-mediated FFA uptake and GLUT4-mediated glucose uptake in response to stimuli share highly conserved components in heart and skeletal muscle [36]. We recently demonstrated that the exocyst subunit EXOC5 is recruited to GLUT4 vesicles in response to insulin and that the exocyst has a role in insulin-induced GLUT4 membrane delivery and subsequent glucose uptake in skeletal muscle cells [22]. To determine if, like in GLUT4 trafficking, the exocyst is recruited to CD36 vesicles as well, we performed PLAs to assess the subcellular proximity of exocyst subunit EXOC5 with CD36 after insulin and ionomycin treatment in the presence or absence of the exocyst inhibitor ES2. Insulin treatment of the L6 GLUT4myc cells stimulated EXOC5 and CD36 association, as indicated by an increased signal-to-nuclei ratio (Figure 1F). In addition, the presence of the exocyst inhibitor ES2 hindered exocyst subunit recruitment to the CD36 vesicles in response to insulin. Following ionomycin treatment, L6 GLUT4myc cells showed a similar increase in EXOC5 and CD36 proximity (Figure 1G). However in the presence of the inhibitor ES2, ionomycin treatment failed to trigger a comparable change in EXOC5 and CD36 association. Endosidin-2 treatment similarly affects insulin-induced exocyst recruitment to GLUT4 vesicles in skeletal myoblasts [22]. Recent interaction models distinguish two exocyst subcomplexes, consisting of EXOC1–4 (subcomplex I) and EXOC5–8 (subcomplex II) [37]. As the inhibitor ES2 targets the EXOC7 subunit of subcomplex II [33], our data indicate that subcomplex II assembly is necessary for the association of the Exoc5 subunit with these fuel transporter-vesicles upon physiological stimuli.

### 3.3. Disrupted Exocyst Function Impairs CD36 Membrane Delivery and FFA Uptake in Response to Stimuli

The study that identified the exocyst as a key regulator of insulin-stimulated FFA uptake [18] also revealed that siRNA-mediated silencing of the EXOC7 and EXOC8 exocyst subunits significantly reduced FFA uptake under both basal and insulin-treated conditions compared to controls. Exocyst activity can be modulated by targeting EXOC5, its central subunit, as EXOC5 silencing in cell culture and animal models causes protein degradation of several other exocyst members [38,39,40].

Thus, we assessed the effect of impaired exocyst activity on stimulated CD36 surface delivery and subsequent FFA uptake by ES2 inhibitor treatment and by heterozygous EXOC5 knockout (ΔEXOC5) in L6 GLUT4myc skeletal myoblasts. (Generation and characterization of the ΔEXOC5 clones were published earlier by our group [22].) As exocyst function is necessary for the proper differentiation of skeletal myoblasts into myotubes, we used undifferentiated ΔEXOC5 and wild-type L6 GLUT4myc myoblasts as respective controls for some of our experiments. We applied an in-cell Western approach, where a quantitative immunofluorescence assay directly detects CD36 surface protein levels in cells grown in multiwell plates with a primary antibody that recognizes the extracellular domain of CD36. This fluorescence-based in-cell Western surface detection of proteins is built on methods adapted from Wang et al. [41] to quantify glucose transporter GLUT4 surface delivery in skeletal myoblasts. We measured changes in cell surface CD36 delivery in our cultured wild-type and ΔEXOC5 myoblasts and control or ES2-treated myoblasts and differentiated myotubes.

**Figure 2:**
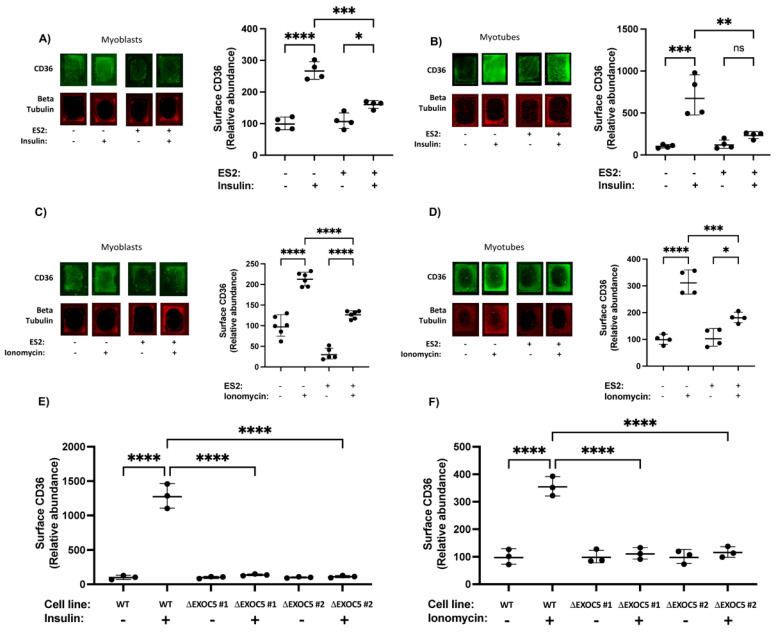
Disrupted exocyst function impairs CD36 membrane delivery. Representative in−cell Western analysis shows that insulin stimulates higher cell−surface levels of CD36 in L6 GLUT4myc myoblasts (**A**) and differentiated myotubes (**B**), an effect significantly reduced in the presence of the exocyst inhibitor ES2. Signal intensities of the entire surface of the chamber slide wells were measured with a fluorescent scanner. Surface CD36 levels were normalized to β−tubulin levels measured following permeabilization and immunostaining. Representative in−cell Western analysis and quantitation reveal elevated cell surface CD36 levels in L6 GLUT4myc myoblasts (**C**) and differentiated myotubes (**D**) treated with ionomycin. This response to ionomycin treatment is blunted in the presence of ES2. Quantification of relative surface CD36 levels in wild−type and ΔEXOC5 myoblasts treated with insulin (**E**) or ionomycin (**F**) indicates that exocyst disruption impairs membrane trafficking of the FFA translocase. Data are means ±S.D. in percent ratios relative to the untreated or wild−type controls. * *p* < 0.05; ** *p* < 0.01; *** *p* < 0.005; **** *p* < 0.0005; ns: *p >* 0.05. The images and data presented are representative of three independent measurements.

Upon quantifying cell surface CD36, we found that both insulin and ionomycin significantly increased CD36 surface delivery in rat skeletal myoblasts and differentiated myotubes (Figure 2A,B). ES2 inhibitor treatment hindered the membrane trafficking of CD36 in response to both stimuli (Figure 2A–D). This suggests that disrupting exocyst assembly directly affects the amount of CD36 on the cell surface, which can have implications for FFA uptake and metabolism.

Our CD36 cell surface delivery measurements in wild-type and heterozygous Exoc5 knockout (ΔEXOC5) L6 GLUT4myc clones demonstrate that wild-type skeletal myoblasts have a significant increase in cell surface CD36 following both insulin and ionomycin treatment. However, EXOC5 depletion and, as a consequence, decreased exocyst activity in skeletal myoblasts lead to significantly lower rates of CD36 membrane delivery in response to both insulin and the contraction-mimetic ionomycin (Figure 2E,F).

**Figure 3:**
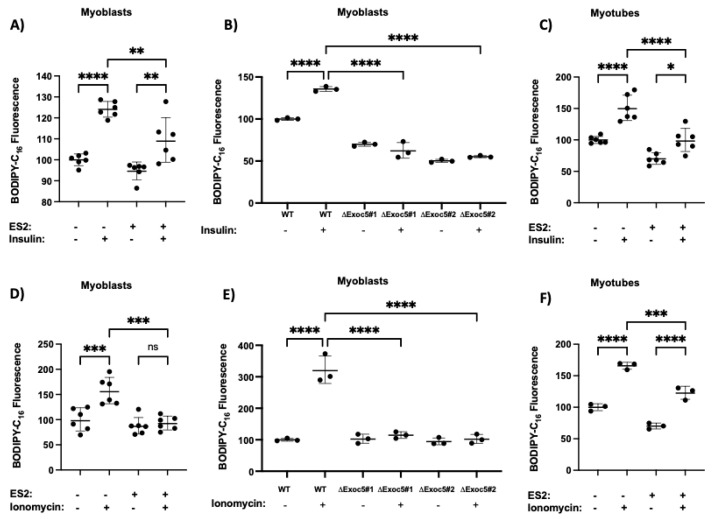
Disrupted exocyst function hinders FFA uptake in response to stimuli. Measurement of fluorescent long−chain FFA analog BODIPY−C16 uptake in wild−type (**A**) and ΔEXOC5 skeletal myoblasts (**A**,**B**) and differentiated myotubes (**C**) in response to insulin reveals that impaired exocyst function hinders FFA uptake. Measurement of fluorescent long−chain FFA analog BODIPY−C16 uptake in wild−type (**D**) and ΔEXOC5 skeletal myoblasts (**E**) and differentiated myotubes (**F**) in response to ionomycin treatment demonstrates that defective exocyst activity impedes FFA uptake in this model. Data are means ±S.D. in percent ratios relative to the untreated or wild−type controls. * *p* < 0.05; ** *p* < 0.01; *** *p* < 0.005; **** *p* < 0.0005; ns: *p >* 0.05. Data presented are representative of three independent measurements.

Skeletal muscle FFA uptake in response to physiological stimuli is proportional to the amount of CD36 on the cell surface, and other fatty acid transporters (FATP1, FATP4 and FABP) do not contribute significantly to this process. As CD36 membrane trafficking was affected by exocyst disruption, we next examined FFA uptake in skeletal myoblasts and differentiated myotubes by measuring fluorescently-labeled long-chain FFA palmitate (BODIPY-C16) uptake. Like natural and radio-labeled fatty acid analogs, the BODIPY-labeled FFAs enter the cell and are efficiently incorporated into the triglyceride pool in 3T3-L1 adipocytes [42]. In response to both insulin and ionomycin treatment, we detected a robust accumulation of BODIPY-C16 in wild-type myoblasts and myotubes (Figure 3). Neither stimulus could trigger such a response in myoblasts or myotubes in the presence of exocyst inhibitor ES2, consistent with a disrupted cell surface delivery of CD36. Similarly, ΔEXOC5 skeletal myoblasts failed to increase FFA uptake in response to both insulin and ionomycin treatment compared to wild-type control myoblasts (Figure 3B,E). While skeletal myoblasts primarily rely on glycolysis, upon differentiation, myotubes shift toward oxidative phosphorylation [43]. However, we did not observe a significant increase in stimulus-induced FFA uptake rates in differentiated myotubes compared to myoblasts.

### 3.4. Rab GTPases Associate with the Exocyst and CD36 in Response to Stimuli

From early studies of the exocyst, we know that all exocyst genes are expressed in skeletal muscle [44,45]. However, almost no studies investigated the exocyst’s biological functions or their regulation in skeletal muscle tissues. Previous reports showed that, in skeletal muscle, the GTPase activating proteins (GAPs) TBC1D1 and Akt substrate 160 (AS160 or TBC1D4), which negatively regulate exocyst-associated Rab GTPases, also participate in GLUT4 trafficking upon insulin induction and AMPK pathway activation [46,47]. In addition, the Rab GAP AS160 mediates insulin and AMPK-stimulated surface translocation of CD36 in cardiomyocytes as well, as knockdown of AS160 redistributes CD36 to the surface and abolishes its membrane delivery by insulin or AMPK activation [20].

**Figure 4:**
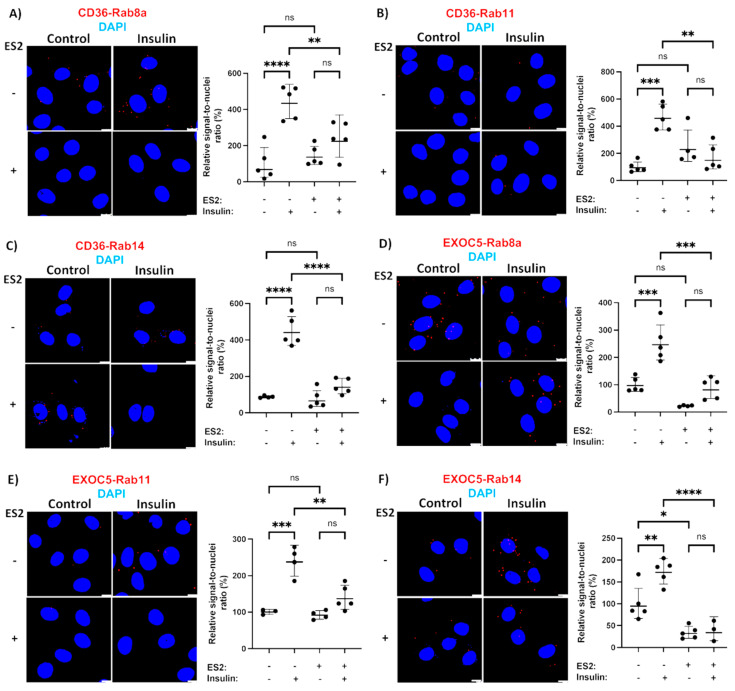
Identifying Rab GTPases associated with the exocyst and CD36 in response to insulin. (**A**–**C**) Microscopic images and quantification of a PLA evaluating proximity of Rab8a, Rab11 and Rab14 with CD36 in L6 GLUT4myc myoblasts show higher association rates of all three Rabs with CD36 following insulin treatment. In the presence of ES2, insulin fails to trigger such an increase in target protein proximity. (**D**–**F**) Microscopic images and quantification of a PLA evaluating proximity of Rab8a, Rab11 and Rab14 with EXOC5 in L6 GLUT4myc myoblasts following insulin treatment demonstrate Exoc5 recruitment to Rabs 8a, 11 and 14. This recruitment is inhibited by treatment with ES2. PLA signals are shown in red; DAPI stain is in blue. Images are representative of three independent experiments. Scale bars: 10 μm. Data are presented as means ± S.D normalized to controls. * *p* < 0.05; ** *p* < 0.01; *** *p* < 0.005; **** *p* < 0.0005; ns: *p >* 0.05.

**Figure 5:**
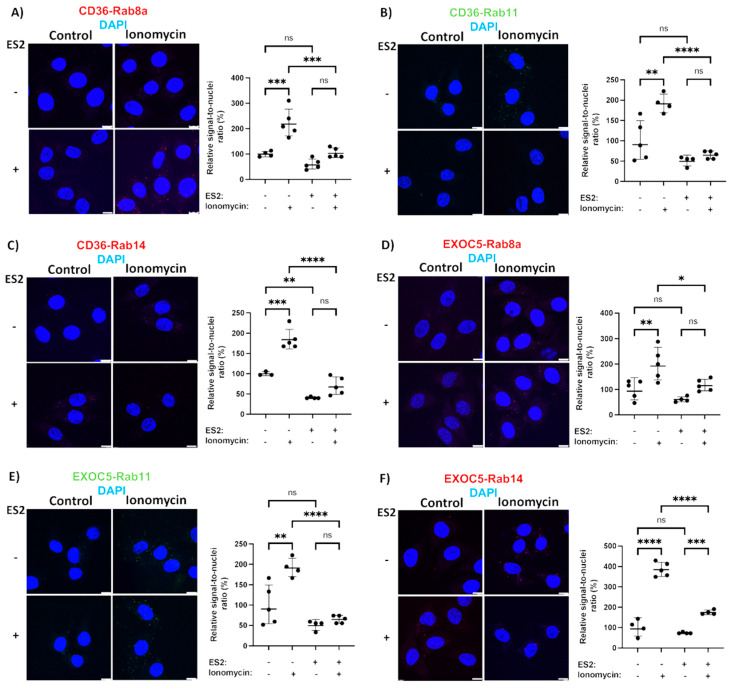
Identifying Rab GTPases associated with the exocyst and CD36 in response to contraction-mimetic treatment. (**A**–**C**) Microscopic images and quantifications of PLAs demonstrate increased proximity of Rab8a, Rab11 and Rab14 with CD36 in L6 GLUT4myc myoblasts following ionomycin treatment. This effect of ionomycin was diminished in the presence of the ES2 inhibitor. (**D**–**F**) Microscopic images and quantifications of PLAs evaluating proximity of EXOC5 with Rab8a, Rab11 and Rab14 in L6 GLUT4myc myoblasts in response to ionomycin treatment reveal elevated signal-to-nuclei ratios, indicating a rise in physical proximity of the target proteins. Exocyst inhibition by ES2 blocks this ionomycin-stimulated association of Exoc5 with Rabs 8a, 11 and 14. PLA signals are shown in red or green; DAPI stain is in blue. Images are representative of three independent experiments. Scale bars: 10 µm. Data are presented as means ± S.D normalized to controls. * *p* < 0.05; ** *p* < 0.01; *** *p* < 0.005; **** *p* < 0.0005; ns: *p >* 0.05.

The Rab proteins responsible for fuel transporter trafficking are recruited and directly bind to the secretory vesicles and their downstream effectors. To identify the Rab GTPases responsible for CD36 trafficking, we used a PLA-based test to evaluate the recruitment of small GTPases to both the CD36 vesicles and the exocyst complex. A similar method identified the regulators of the exocyst activity in bladder epithelium cells [48]. We focused on Rab GTPases that are either known substrates of AS160 (Rab8, Rab14) [49,50] or have shown colocalization with GLUT4 and CD36 vesicles in muscle cells (Rab11) [51]. Based on our results, all three of the Rab proteins studied showed a low level of association with both the CD36 vesicles and the exocyst complex under basal conditions (Figure 4). PLAs also revealed that insulin and ionomycin treatment resulted in a significantly increased recruitment of Rabs 8, 11 and 14 to CD36 vesicles (Figure 4A–C and Figure 5A–C). We observed higher association rates between all three Rabs and the exocyst subunit EXOC5 following both insulin and ionomycin treatment, but exocyst inhibition by ES2 led to significantly lower rates of Rab recruitment in response to stimuli (Figure 4D–F and Figure 5D–F).

However, in the presence of ES2, both treatments failed to trigger a similar rise in Rab GTPase association with both EXOC5 and CD36, indicating that Rabs 8, 11 and 14 may use the exocyst complex as their effector during insulin- and ionomycin-induced CD36 trafficking in skeletal muscle cells. In agreement with our findings, the GTPase Rab8a, a known regulator of exocyst activity, was shown to be essential for insulin-induced CD36 trafficking and FFA uptake in muscle [19,20]. Of note, both Rab8a and Rab14 colocalize with CD36 vesicles in cardiomyocytes and their overexpression results in increased surface CD36 levels. This effect appears to be indirect for Rab14, as its knockdown does not impact CD36 membrane delivery in response to stimuli [20]. Rab11, found primarily on recycling endosomes, was the first small GTPase identified on CD36 vesicles, and it was initially shown to mediate CD36 internalization [52]. This is in line with evidence that recycling endosomes act as an intracellular storage compartment for CD36 [53]. Recent reports describe how Rab11 is necessary for exocyst-mediated recycling endosome exocytosis [54]. Given that the exocyst may serve as a coordinator of endo- and exocytosis [55], future studies aimed at determining the exact role of Rab11 in CD36 trafficking are warranted.

Since CD36 plays a key role in the development of insulin resistance starting from the early stages of intramyocellular lipid accumulation, recent investigations targeted the fatty acid translocase for metabolic intervention. In support of this approach, whole-body and skeletal muscle-specific disruption of CD36 in healthy mice resulted in a significant increase in insulin-mediated glucose uptake, and in high-fat diet-fed mice, CD36 deficiency improved muscle glucose uptake and insulin sensitivity due to a reduced FFA uptake [6,14]. Directly decreasing CD36 translocation to the cell membrane of metabolic tissues could thus provide a mechanism to overcome lipid accumulation leading to insulin resistance in obese and diabetic patients.

To date, several proteins have been reported to facilitate CD36 membrane trafficking, and the exocyst was reported to play a central role in FFA uptake in cultured adipocytes. Our findings reveal that the exocyst regulates FFA uptake via targeted delivery of the FFA translocase CD36 to the muscle cell membrane in response to physiological stimuli. The exocyst is also responsible for coordinating insulin-induced GLUT4 membrane delivery in skeletal muscle tissues, representing a shared component of the membrane trafficking machinery delivering fuel transporters. As exocyst activity is orchestrated by regulatory small GTPases, it is possible that the complex coordinates CD36 and GLUT4 delivery in metabolic tissues via selective trafficking of the transporters guided by its interactions with Rab GTPases found on the secretory vesicles. Our results indicate that the small GTPases Rab8a, 11 and 14 likely play a role in CD36 surface delivery in skeletal muscle. Further studies aimed at the identification of unique exocyst-regulating GTPases necessary for selective CD36 trafficking could provide a possible target to overcome intramyocellular lipid accumulation and the subsequent development of skeletal muscle insulin resistance.

## Data Availability

Not applicable.
